# Rhabdomyolysis in a Patient With Coronavirus Disease 2019

**DOI:** 10.7759/cureus.8956

**Published:** 2020-07-01

**Authors:** Aveek Mukherjee, Raisa Ghosh, Ghulam Aftab

**Affiliations:** 1 Internal Medicine, Rutgers Robert Wood Johnson Medical School/Saint Peter's University Hospital, New Brunswick, USA; 2 Pulmonary Medicine, Rutgers Robert Wood Johnson Medical School/Saint Peter's University Hospital, New Brunswick, USA

**Keywords:** rhabdomyolysis, covid-19, 2019-ncov, sars-cov-2 (severe acute respiratory syndrome corona virus 2), coronavirus, acute kidney injury, creatine kinase, creatine phosphokinase

## Abstract

Coronavirus disease 2019 has rapidly enveloped the world in a pandemic after emerging in Wuhan, China, in December 2019. We describe a 49-year-old man presenting with fever, cough, dyspnea, and myalgia diagnosed with coronavirus disease 2019 along with rhabdomyolysis and acute kidney injury. The creatine phosphokinase was elevated to 23,800 U/L before trending down to normal levels. Rapid identification and treatment with aggressive intravenous hydration and correction of electrolyte abnormalities remain key to successful management. In a pandemic, often atypical presentations of this new disease have to be considered as differentials for early diagnosis and treatment of life-threatening conditions.

## Introduction

Emerging in December 2019 in Wuhan, China, the coronavirus disease 2019 (Covid-19) has rapidly attained pandemic proportions. Caused by a novel beta coronavirus, Covid-19 can present with a multitude of symptoms, with fever, cough, fatigue, and myalgia being very common [1]. We describe a patient with Covid-19, who was found to have rhabdomyolysis with severely elevated creatine phosphokinase (CPK) and acute kidney injury (AKI). We treated the patient with supplemental oxygen, intravenous fluids, and hydroxychloroquine, and he experienced an uneventful recovery.

## Case presentation

**Figure 1:**
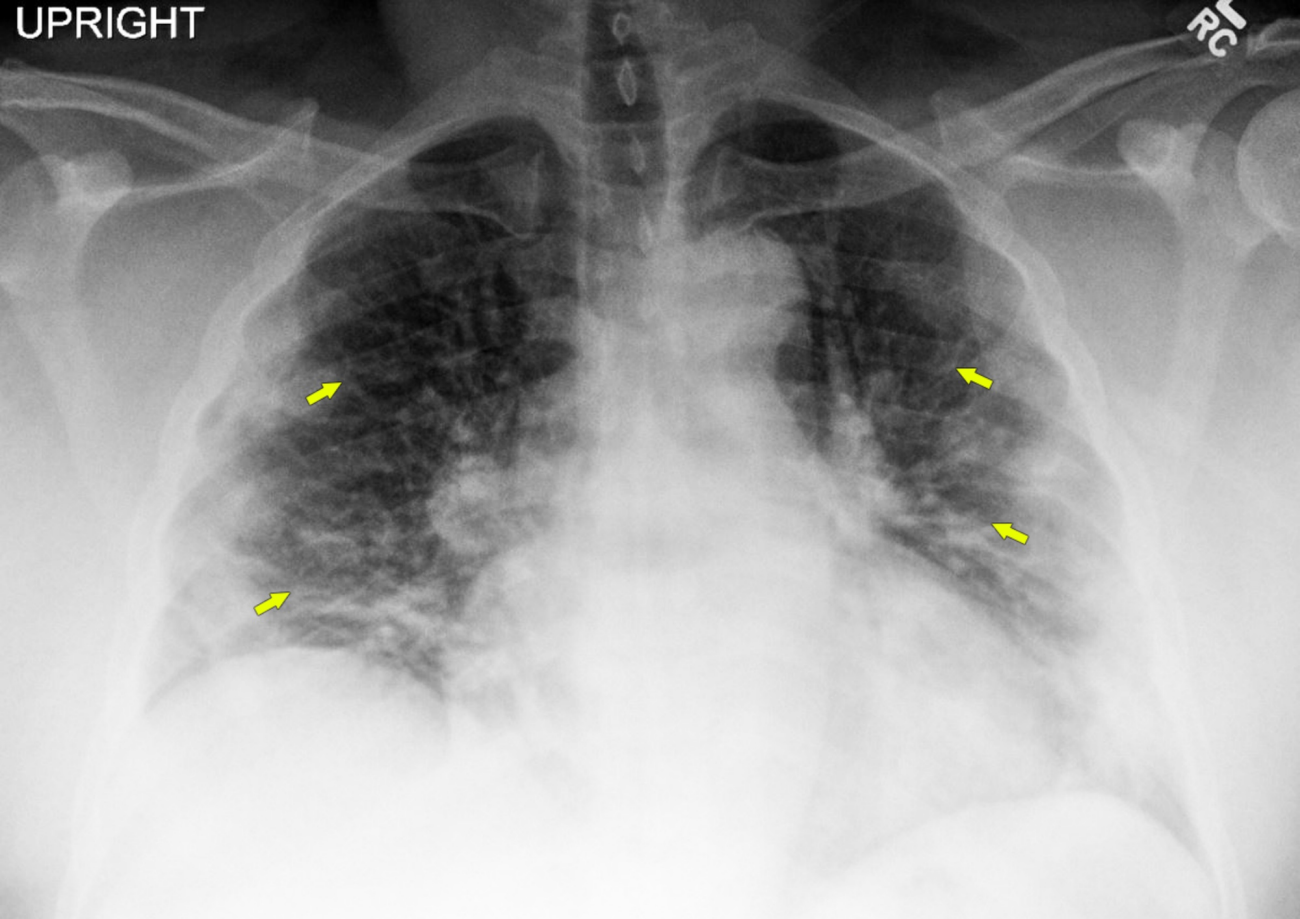
Chest x-ray showing bilateral patchy peripheral infiltrates (arrows).

A 49-year-old man with a history of hypertension and diabetes presented with a week of fever, chills, cough, dyspnea, and intense myalgia. The patient worked as a nurse in a community nursing home, which was likely the exposure to infection. Examination revealed a well-built individual with a body mass index of 46.3 kg/m^2^, who was febrile to 103°F, tachycardic (110 beats/minute), tachypneic (22 breaths/minute), and hypoxic at 88% saturation on room air, without any significant pulmonary examination findings. Initial investigations revealed elevated C-reactive protein (117 mg/L), lactate dehydrogenase (955 U/L), and D-dimer (651 ng/mL), and low absolute lymphocytes (0.91 x 10^9^/L). His chest X-ray revealed bilateral patchy opacities (Figure 1). The overall presentation was suspicious for Covid-19, and the patient was put in an isolation room for treatment per institutional protocols. Subsequently, Covid-19 was confirmed by reverse transcriptase polymerase chain reaction (RT-PCR) assay for severe acute respiratory syndrome coronavirus 2 (SARS-CoV-2). The patient was put on supplemental oxygen via nasal cannula and hydroxychloroquine therapy was initiated.

CPK was found to be extremely high at 22,740 U/L, along-with elevated creatinine at 1.18 mg/dL. Aspartate transaminase (AST), alanine transaminase (ALT), and troponin-I were also elevated (Table 1). His urinalysis revealed large blood and protein without any red blood cells, which was suspicious for myoglobinuria.

**Table 1:** Laboratory parameters with trends by day of hospital admission eGFR, estimated glomerular filtration rate; ND, not done; RBC, red blood cell; WBC, white blood cell

Parameter	Reference value or range	Day 1	Day 2	Day 3	Day 4	Day 5	Day 6	Day 7	Day 8
Hemoglobin (g/L)	120-160	120	115	ND	112	110	111	115	115
White blood cell count (x10^9^/L)	4-11	9.1	8.6	ND	10.9	7.7	7.2	6.9	6.6
Absolute lymphocyte count (x10^9^/L)	1-3.5	0.91	1.72	ND	ND	ND	1.73	1.52	1.58
Platelets (x10^9^/L)	150-400	161	162	ND	205	262	370	437	316
D-Dimer (ng/mL)	0-211	651	ND	ND	ND	ND	ND	499	ND
Lactate dehydrogenase (U/L)	140-271	955	ND	ND	ND	ND	ND	630	ND
C-reactive protein (mg/L)	0-5	117	ND	ND	ND	ND	ND	51	ND
Creatine phosphokinase (U/L)	55-170	22,740	23,060	23,800	15,240	7,210	2,941	1,498	944
Alanine aminotransferase (U/L)	21-72	160	133	135	ND	ND	ND	ND	ND
Aspartate aminotransferase (U/L)	17-59	470	424	411	ND	ND	ND	ND	ND
Troponin-I (ng/mL)	<0.03-0.12	0.12, 0.12	0.08	ND	ND	ND	ND	ND	ND
Blood urea nitrogen (mg/dL)	6-20	13	12	10	10	9	8	8	8
Creatinine (mg/dL)	0.66-1.1	1.18	1.14	0.96	0.96	0.86	0.86	0.76	0.72
eGFR (mL/min/1.73 m^2^)	>60	65	68	83	83	94	94	109	116
Sodium (mmol/L)	136-145	127	127	129	136	142	139	137	137
Potassium (mmol/L)	3.5-5.1	3.2	3.1	3.0	3.3	3.7	3.7	3.4	3.8
Magnesium (mEq/L)	1.3-2.2	ND	1.5	1.7	1.7	1.3	1.1	1.3	1.7
Calcium (mg/dL)	8.4-10	7.9	7.6	7.4	7.2	7.7	7.8	8.0	8.4
Bicarbonate (mmol/L)	21-33	32	29	32	28	29	33	34	33
Phosphate (mg/dL)	2.7-4.5	2.1	ND	2.2	ND	2.9	ND	ND	3.1
Urinalysis		Clear, specific gravity 1,013, pH 6.5, large blood, protein >500, 0-2 RBC, 0-5 WBC	ND	Clear, specific gravity 1,004, pH 6.5, large blood, protein 100, 0-2 RBC, 0-2 WBC	ND	ND	ND	ND	ND

**Figure 2:**
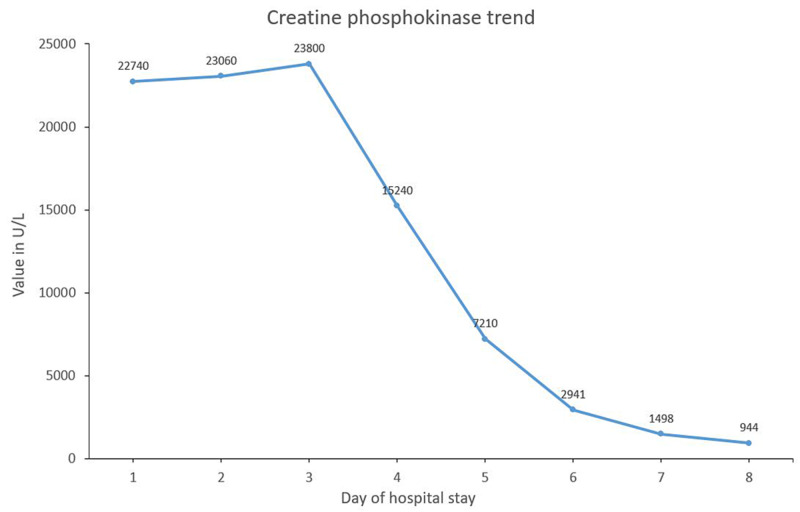
Creatine phosphokinase trend during hospital stay X-axis: day of hospital stay; Y-axis: creatine phosphokinase value in U/L

We suspected AKI secondary to rhabdomyolysis in this clinical setting. Bolus intravenous fluids were immediately transfused followed by maintenance fluids at a rate sufficient to maintain good urine output. Investigations were repeated to follow trends (Table 1, Figure 2).

Further history revealed no illicit drugs or alcohol use. An influenza A/B assay was negative. A search for other etiologies of rhabdomyolysis was unrevealing. The patient was additionally noted to have hyponatremia and hypokalemia which were corrected. Fevers abated by day 3 along with some resolution of myalgia allowing the patient to ambulate in the room. However, due to a transient worsening of dyspnea on day 6, we discontinued intravenous fluids and diuresed with intravenous furosemide. The patient responded favorably with resolution of the myalgia and CPK trending down to 944 U/L. Over the next two days, he was also successfully weaned off the supplemental oxygen. The patient was now feeling significantly better with the resolution of his myalgia and dyspnea. The patient was discharged home after eight days of hospitalization.

## Discussion

Rhabdomyolysis occurs due to the rapid breakdown of skeletal muscle leading to leakage of toxic cellular elements. It can result from direct myocyte injury or failure of energy production, leading to an unregulated increase in intracellular calcium and cellular lysis [2-4]. Etiology includes trauma, exertion, chemicals including medications and drugs, myopathies and metabolic syndromes as well as infections [3,4]. Infectious myositis may be caused by numerous microbes and can occasionally result in rhabdomyolysis [5]. Myalgias frequently present with the acute viral prodromes; however, it is more common in children than adults. It is generally self-limiting, but persistent myalgia and weakness should raise concern for underlying rhabdomyolysis. A wide range of viruses have been implicated in myositis and virus-induced rhabdomyolysis. Influenza A and B are commonly associated, while others such as enteroviruses, human immunodeficiency virus, cytomegalovirus, Epstein-Barr virus, and herpes simplex virus are less common [5-7]. Recently, the severe acute respiratory syndrome coronavirus (SARS-CoV) has also been associated with rhabdomyolysis [8,9]. Rhabdomyolysis has been infrequently reported in patients with Covid-19 [1,10].

The pathogenesis of virus-induced rhabdomyolysis is still unclear, although several mechanisms have been proposed. Viruses could directly invade the myocytes, as seen in influenza and SARS-CoV infections [5-7]. Immunological mechanisms likely play a major role as well. Immune cross-reactivity between viral antigens and myocytes, “innocent-bystander” damage from the deposition of antigen-antibody complexes, and possible viral transformation of the host cell or “haptenization” of host proteins may lead to a dysregulated immune response and muscle damage [5]. A heightened immunologic reaction resulting in “cytokine storm” can further lead to disseminated tissue damage including rhabdomyolysis [7,11]. Cytotoxic T-cell-mediated attack has also been demonstrated to cause muscular injury in viral myositis [12]. Our knowledge of the novel disease, Covid-19, is still evolving, and the exact mechanism of rhabdomyolysis due to SARS-CoV-2 is still unknown. In our patient, we hypothesized that the rhabdomyolysis was likely secondary to SARS-CoV-2 induced myositis in the absence of other etiologies.

AKI is the acute worsening of renal function, as defined by the criteria set by the Kidney Disease Improving Global Guidelines (KDIGO) [13,14]. The classification and treatment of AKI also follow KDIGO guidelines. AKI is a common complication of rhabdomyolysis, accompanying 7%-10% of cases [2]. The presence of AKI leads to increased morbidity and mortality in patients with rhabdomyolysis [2,3]. Rhabdomyolysis results in explosive leakage of myocyte contents, such as myoglobin, electrolytes, and other sarcoplasmic proteins, such as CPK, aldolase, lactate dehydrogenase, ALT, and AST [2-4]. The myoglobin induces renal tubular injury as a result of intrarenal vasoconstriction, direct and ischemic tubular injury, and tubular obstruction [2]. Renal vasoconstriction is a characteristic feature in these cases. It results from intravascular fluid depletion due to sequestration in damaged muscle and release of endogenous mediators resulting in reduced renal blood flow, such as endothelin-1, thromboxaneA_2_, tumor necrosis factor α, and F_2_-isoprostanes. These collectively result in the development of AKI.

Less than 10% of patients with rhabdomyolysis present with the classical triad of weakness, myalgia, and tea-colored urine; hence clinical suspicion and close monitoring are cornerstones for diagnosing rhabdomyolysis [2-4]. Often ALT and AST are also elevated, though AST has a much higher concentration in muscles [4]. CPK elevations five to ten times of normal is diagnostic of rhabdomyolysis [3,4]. Management is usually multipronged. Intravenous rehydration to ensure urine output of 200-300 mL/hour (3 mL/kg of body weight per hour) is key to management [2-4]. Often patients may need to be transfused up to 10 L of fluids daily, depending on clinical features [2]. As dyselectrolytemia is often responsible for worse clinical outcomes, it should be corrected promptly and then monitored. The administration of sodium bicarbonate is recommended when metabolic acidosis is present concurrently and urine pH is less than 6.5. Furthermore, clinical improvement of the patient and normalization of laboratory parameters are also important markers. We treated our patient with aggressive intravenous fluids to maintain the recommended urine output, while concurrently correcting electrolyte disorders. For treatment of the hypoxia, the patient was started on supplemental oxygen therapy which was slowly weaned over days. We also treated the patient with hydroxychloroquine for Covid-19 according to our institutional protocols [15].

## Conclusions

Rhabdomyolysis, especially in association with AKI, carries high morbidity and mortality. It is often not evident in the initial presentation. In the setting of a pandemic and due to infrequent presentation, we suggest that clinicians consider rhabdomyolysis as a differential for AKI in patients with Covid-19 to be able to diagnose and treat such life-threatening conditions early. Aggressive intravenous hydration, correction of dyselectrolytemia, and monitoring laboratory markers are key to its management.

## References

[1] Guan W-Z, Ni Z-Y, Hu Y (2020). Clinical characteristics of coronavirus disease 2019 in China. N Engl J Med.

[2] Bosch X, Poch E, Grau JM (2009). Rhabdomyolysis and acute kidney injury. N Engl J Med.

[3] Zutt R, van der Kooi AJ, Linthorst GE, Wanders RJA, de Visser M (2014). Rhabdomyolysis: review of the literature. Neuromuscul Disord.

[4] Stanley M, Adigun R (2020). Rhabdomyolysis.. http://www.ncbi.nlm.nih.gov/books/NBK448168/.

[5] Crum-Cianflone NF (2008). Bacterial, fungal, parasitic, and viral myositis. Clin Microbiol Rev.

[6] Ayala E, Kagawa FT, Wehner JH, Tam J, Upadhyay D (2009). Rhabdomyolysis associated with 2009 influenza A(H1N1). JAMA.

[7] Fadila MF, Wool KJ (2015). Rhabdomyolysis secondary to influenza a infection: a case report and review of the literature. North Am J Med Sci.

[8] Chen L-L, Hsu C-W, Tian Y-C, Fang J-T (2005). Rhabdomyolysis associated with acute renal failure in patients with severe acute respiratory syndrome. Int J Clin Pract.

[9] Huang J-W, Chen K-Y, Tsai H-B (2005). Acute renal failure in patients with severe acute respiratory syndrome. J Formos Med Assoc.

[10] Jin M, Tong Q (2020). Rhabdomyolysis as potential late complication associated with COVID-19. Emerg Infect Dis.

[11] Liang Y, Wang M-L, Chien C-S (2020). Highlight of immune pathogenic response and hematopathologic effect in SARS-CoV, MERS-CoV, and SARS-Cov-2 infection. Front Immunol.

[12] Pancheri E, Lanzafame M, Zamò A (2019). Benign acute viral myositis in African migrants: a clinical, serological, and pathological study. Muscle Nerve.

[13] Gameiro J, Fonseca JA, Outerelo C, Lopes JA (2020). Acute kidney injury: from diagnosis to prevention and treatment strategies. J Clin Med.

[14] Khwaja A (2012). KDIGO clinical practice guidelines for acute kidney injury. Nephron Clin Pract.

[15] Mukherjee A, Ahmad M, Frenia D (2020). A coronavirus disease 2019 (COVID-19) patient with multifocal pneumonia treated with hydroxychloroquine. Cureus.

